# Cellular Adaptation Takes Advantage of Atavistic Regression Programs during Carcinogenesis

**DOI:** 10.3390/cancers15153942

**Published:** 2023-08-03

**Authors:** Davide Gnocchi, Dragana Nikolic, Rosa Rita Paparella, Carlo Sabbà, Antonio Mazzocca

**Affiliations:** Interdisciplinary Department of Medicine, University of Bari School of Medicine, Piazza G. Cesare, 11, 70124 Bari, Italy; davide.gnocchi@uniba.it (D.G.); dragana.nikolic@uniba.it (D.N.); ritabiotec1@gmail.com (R.R.P.); carlo.sabba@uniba.it (C.S.)

**Keywords:** tumor biology, tumor metabolism, tumor adaptation, cancer drug resistance

## Abstract

**Simple Summary:**

Cancer can be considered the epidemic of the third millennium, and recent projections paint a worrying scenario for the coming decades. Knowledge about the causes of neoplastic transformation remains limited, and classical genetic paradigms do not provide adequate models. Likewise, current pharmacological approaches show limited efficacy and often lead to the development of drug resistance and relapse. The limited success of cancer treatments requires a revision and, possibly, a paradigm shift in how we think about the disease. In this regard, studies of tumor cell metabolism and mechanisms of tumor cell adaptation can provide important insights. Here, we review the latest knowledge on the impact of metabolic and microenvironmental conditions on determining the phenotype of tumor cells and on how increasing this understanding could improve pharmacological options.

**Abstract:**

Adaptation of cancer cells to extreme microenvironmental conditions (i.e., hypoxia, high acidity, and reduced nutrient availability) contributes to cancer resilience. Furthermore, neoplastic transformation can be envisioned as an extreme adaptive response to tissue damage or chronic injury. The recent Systemic–Evolutionary Theory of the Origin of Cancer (SETOC) hypothesizes that cancer cells “revert” to “primitive” characteristics either ontogenically (embryo-like) or phylogenetically (single-celled organisms). This regression may confer robustness and maintain the disordered state of the tissue, which is a hallmark of malignancy. Changes in cancer cell metabolism during adaptation may also be the consequence of altered microenvironmental conditions, often resulting in a shift toward lactic acid fermentation. However, the mechanisms underlying the robust adaptive capacity of cancer cells remain largely unknown. In recent years, cancer cells’ metabolic flexibility has received increasing attention among researchers. Here, we focus on how changes in the microenvironment can affect cancer cell energy production and drug sensitivity. Indeed, changes in the cellular microenvironment may lead to a “shift” toward “atavistic” biologic features, such as the switch from oxidative phosphorylation (OXPHOS) to lactic acid fermentation, which can also sustain drug resistance. Finally, we point out new integrative metabolism-based pharmacological approaches and potential biomarkers for early detection.

## 1. Introduction

Interactions between tumor cells and the physicochemical conditions of the tumor microenvironment (TME) regulate tumor cell proliferation, metabolism, and response to therapy. The TME can be described in terms of the cellular (e.g., stromal cells and infiltrating immune cells) and molecular determinants (e.g., cytokines, metabolites, exosomes, and extracellular matrix) with which solid tumor cells are surrounded and to which they respond. On the one hand, this complex TME enables tumor cells to maintain evasion strategies to thrive in a challenging environment [1], and on the other hand, the TME contributes to the metabolic rewiring of cancer cells. In particular, effects on lipid homeostasis have revived interest in cancer metabolism [2]. In addition, very often, the non-genetic mechanisms are linked to the genetic landscape of the tumor in escaping therapy through adaptive processes (changes in the cancer cells’ transcriptional and/or metabolic program) and/or cellular plasticity, which enables the evasiveness of anticancer immunosurveillance [3]. Thus, cellular adaptation and plasticity have emerged as new challenges in the field. Recently, the scientific community has also begun to explore the mechanisms of non-genetic drug resistance, suggesting that it can be found as “unstable” and “stable”. Regardless, epigenetic heterogeneity and cellular plasticity appear to be relevant factors in the development of drug resistance, which may be driven by interactions between genetic and nongenetic mechanisms. So, cancer cells can adapt through genetic and/or epigenetic mechanisms, thus highlighting the potential interaction between genetic and non-genetic adaptation and underlining the role of environmental factors. However, existing data explain how the TME activates different pathways or alters metabolism to release different metabolites, while data at the mitochondrial level are still limited [4].

Experimental studies have been limited by the difficulty of controlling the fluctuating internal conditions of the TME, such as oxygen and nutrients [5]. Tumor cells divide rapidly, consuming oxygen and nutrients, despite the disorganized vasculature of solid tumors that limit access to a steady supply of nutrients and oxygen from circulation, leading to nutrient starvation, oxygen deprivation, and, hence, the formation of necrotic zones [6]. However, cells in the middle layer (between the proliferative edge and the necrotic zone) enter a quiescent state and adapt to the lack of nutrients and limited oxygen by altering their metabolic activity and increasing metabolic flexibility and resilience. As a result, quiescent tumor cell subsets increase, as seen in many types of cancer [7]. The signaling pathways and exact mechanisms by which cells enter quiescence are not well understood [8]. Hypoxia-induced stress and acidosis in the TME are thought to be the main causes [9]. Cell dormancy is a reversible state under certain stimuli (changes in the microenvironment, such as nutrient restoration or changes in oxygen supply), and dormant cells may re-enter the cell cycle, while the development of necrotic zones is inevitable if nutrient and oxygen supply is restricted for prolonged periods. Consequently, nutrients and oxygen are reduced through the TME, suggesting that a heterogeneous TME influences tumor cell viability and dictates a proliferation gradient [5,6,7]. There is an increasing scientific interest in the metabolic flexibility of cancer cells [10] and in the interplay between tumor cell metabolism and the TME [11,12,13] for the understanding of cancer metabolism and for translational purposes. The identification of non-genetically driven metabolic adaptations that inhibit the effects of drug treatments suggests that metabolic plasticity may confer disadvantages on drug treatments. Studies comparing the metabolism of metastasis-forming breast cancer cells with isogenic non-metastatic cells have revealed differences in lactate production, glucose, and oxygen consumption, leading to changes in cancer fitness and plasticity due to changes in the TME [14].

Here, we summarize current knowledge on microenvironmental conditions, such as hypoxia, nutrient deprivation, and acidic environments, and discuss how these conditions affect mitochondrial activity. We also discuss the role of cellular adaptation during carcinogenesis in the light of the new hypothesis of “atavistic regression” as a model for cellular transformation [15]. This hypothesis has also been envisioned by the Systemic–Evolutionary Theory of the Origin of Cancer (SETOC) [16,17]. As postulated by the SETOC, changes in the cellular microenvironment induce adaptations that lead to uncoupling of the two endosymbiotic cellular subsystems (nucleus and mitochondria). This uncoupling may represent a facilitator of carcinogenesis. The transition from oxidative phosphorylation (OXPHOS) to lactic acid fermentation (Warburg effect) can be considered a metabolic signature of the uncoupling of the two endosymbiotic systems.

Finally, we will discuss how changes in microenvironmental conditions and cellular adaptation are involved in the metastatic process and drug resistance. We will outline what is known and why it is important to conduct more research in this direction. We will also highlight the promising role of nutraceuticals in future therapeutic applications, as they can target entire biological processes rather than single druggable targets.

## 2. Survival under Harsh Environmental Conditions: The Central Role of Mitochondria in Adaptive Mechanisms

Tricarboxylic acids and glycolytic intermediates endow cancer cells with the flexibility to respond adequately to the changing microenvironment during tumor evolution [18]. Mitochondrial metabolism adapts to stressful conditions to maintain the bioenergetic levels required for cellular function. We propose that the reaction of cancer cells to cellular stress is critical for metabolic flexibility and bioenergetic adaptation, as well as for tumor development, progression, and metastasis. Thus, cancer cells have adapted multiple mechanisms to maintain mitochondrial function for survival. In a nutrient- and oxygen-starved environment, tumor cells become nutrient-/oxygen-starved and adapt by reducing their need for ATP, maintaining the ATP/ADP ratio through different mechanisms to maintain viability [19]. Mitochondria are involved in the metabolic rewiring and plastic behavior of cancer cells, play a key role in tumor progression, and represent potential therapeutic targets [20]. Targeting mitochondria metabolism and understanding tumor metabolism may allow the control of tumor development, as well as the circumvention of therapy resistance.

German biochemist Otto Warburg was the first to report that cancer cells preferentially generate energy through fermentative glycolysis or lactic acid fermentation even in the presence of oxygen, rather than using oxidative phosphorylation (OXPHOS) as healthy cells do. The importance of this metabolic switch towards lactic acid fermentation in cancer cells, also referred to as the “the Warburg effect”, has recently been recognized again after decades of cancer research that has mainly been focused on genetics [21,22]. In fact, it has been shown that the Warburg effect is primarily triggered by mitochondrial defects. In particular, after conversion into pyruvate, glucose is reduced to lactate-by-lactate dehydrogenase A (LDHA) instead of being transported to the mitochondria. This response is stimulated by hypoxia-inducible factor 1 (HIF-1), which regenerates the reduced form of nicotinamide adenine dinucleotide (NADH) to NAD^+^ [20]. NAD^+^ and H^+^ are then secreted into the microenvironment via the monocarboxylate transporter (MCT), resulting in a decrease in the pH of the extracellular environment (pHe) and an increase in lactate concentration. These metabolic conditions contribute to tumorigenesis and favor the acquisition of metastatic properties. In addition, some cancer cells take up lactate and use it as fuel for mitochondrial oxidative metabolism, converting it back into pyruvate with lactate dehydrogenase B (LDHB). This effect is called “the reverse Warburg effect” [23,24]. Given the differences in oxygen concentration gradients in the tumor burden [13], it has been proposed that glycolytic cells work in concert with oxidative cells to regulate access to energy metabolites: Glycolytic cells export lactate to the TME, while oxidative tumor cells import it [25]. However, in breast cancer, increased lactate uptake occurs only in tumor areas where oxygen is present [26]. Several studies support that circulating lactate contributes to the production of TCA cycle intermediates in cancer cells [24,27]. Hence, according to this view, glycolysis and OXPHOS in tumors are not mutually exclusive but are both required for tumor cell growth and maintenance. Cancer cells can remodel their metabolism to meet demands for energy and biosynthetic precursors. Emerging evidence seems to support this notion, as only double knockdown of the *LDHA/B* gene abolishes lactate production and rewires cancer metabolism to OXPHOS to evade death. Furthermore, this event reduces tumor growth, which becomes more sensitive to mitochondrial complex I inhibitor phenformin [28].

Metabolites produced by glycolysis and mitochondrial metabolism act as substrates and signaling molecules for chemical reactions [29]. In addition, these metabolites can inhibit enzymes through competitive inhibition or post-translational modification [30,31]. These functions are mainly determined by their intracellular and extracellular concentrations and their sub-compartmental localization [29,30]. Notably, the signaling functions of these metabolic intermediates go beyond self-regulation to include cellular communication and sensing of microenvironmental conditions to elicit stress responses and cellular adaptation. Upregulation of glutamine uptake and metabolism is considered a second important metabolic shift in tumor cells [32,33].

OXPHOS has also emerged as a potential source of tumor biomarkers with clinical value. Altered expression and/or mutations of mitochondrial complex IV (CIV) subunits have been found in some forms of cancer. Inhibition of CIV reduces cell proliferation in the glioma and glioblastoma [34]. However, mutations leading to the loss or reduction of CIV activity, thereby promoting the upregulation of glycolysis, stimulated growth and invasion in colorectal and esophageal cancers [35,36]. Mitochondrial complex III (CIII) and, especially, its ubiquitin cytochrome c reductase-binding protein (UQCRB) subunit may be a prognostic marker in colorectal cancer. UQCRB is upregulated at both the gene and protein levels in colorectal cancer tissues compared with non-tumor tissues. In addition, incremental and copy number genetic variations are associated with the clinical stage and pathologic features [37]. CIV, along with CI, CIII, and ATP synthase, is encoded by nuclear and mitochondrial genes. The ratio between nuclear-/mitochondrial-encoded genes of CIV was similar to that in prostate tumor progression. Cancers of the ovary, colon, esophagus, and breast showed the same trend. This transition may be a valuable biomarker that can predict tumor evolution even before histological changes [38]. These data highlight how small defects in the electron transport chain (ETC) modify mitochondrial activity, predisposing cells to different bioenergetic profiles or redox states. These adaptations can act as potential pro- or anti-tumor drivers. Hence, the role of mitochondria in a specific TME and specific alterations to normal function should be considered to predict potential transformation. However, functional ETC sustains OXPHOS activity, which promotes cell differentiation, suggesting the idea of restoring mitochondrial respiration as a possible strategy for reducing cancer cell growth [39].

Early diagnosis remains one of the major challenges in cancer treatment, as diagnostic methods are often invasive or lack specificity/sensitivity. Changes in mitochondrial metabolism and apoptosis during tumorigenesis highlight the relevance of mitochondria in malignancy and serve as potential tumor biomarkers that could improve tumor screening and aid in treatment selection. For example, Lisanti’s group, by using bioinformatics, identified nuclear-encoded mitochondrial genes that were useful as tumor biomarkers for non-small-cell lung cancer [40]. Collectively, mitochondrial biomarkers can predict the risk of ovarian, lung, or breast cancer in high-risk cancer patients for up to 5, 10, or 15 years, respectively [40,41,42].

## 3. Hypoxia and Adaptive Mechanisms in Cancer Metabolism

Hypoxia is an important aspect of tumor cell metabolism and adaptive mechanisms. As recognition for their discoveries on how cells sense and adapt to oxygen availability through the HIF signaling pathway, William G. Kaelin Jr., Sir Peter J. Ratcliffe, and Gregg L. Semenza were awarded the Nobel Prize in Physiology or Medicine in 2019. Inadequate vascularization is a major cause of cancer hypoxia, which is often a combination of acute (perfusion hypoxia), chronic (diffuse hypoxia), and systemic forms of hypoxia. Hypoxia was associated with gene reprogramming of hypoxia-inducible transcription factors (HIFs) and with a more aggressive phenotype due to an immunosuppressive microenvironment and to a promotion of cancer cell metabolism [43]. HIF-1α protein is stabilized at low O_2_ levels, a condition that favors its accumulation, and then transported to the nucleus [44], where it regulates the expression of several genes involved in glycolysis, pH regulation, angiogenesis, and wound healing. However, hypoxia can also be activated through oncogene-driven signaling pathways independent of oxygen supply. Increased glucose consumption, inhibition of the ETC, and incomplete activity of the TCA cycle are observed during hypoxia (or pseudohypoxia). These events correlate with alterations in the levels of several metabolites (lactate, fumarate, succinate, citrate, etc.), which promote tumor development and/or contribute to the stabilization of HIF-1α, thus exacerbating hypoxia-mediated signaling pathways [45]. Pyruvate mediates the adaptive response to hypoxia, as its conversion into lactate is necessary to maintain cellular homeostasis in the hypoxic state. Thus, elevated pyruvate levels promote HIF-1α stabilization and glycolysis, stimulate angiogenesis, and inhibit apoptosis. Pyruvate dehydrogenase (PDH) is regarded as a metabolic target because it promotes hypoxia through HIF-1α stabilization and lactate production. The fact that lactate favors cancer development and progression, as well as drug resistance, makes this metabolite a central target for therapies [45].

Adaptive mechanisms for cancer hypoxia that drive tumor progression are recognized as a major cause of resistance to various therapies [44,46]. One potential pathway of drug resistance is the metabolic adaptation of hypoxic cancer cells to HIF-1α inhibition. Accumulating evidence suggests that adaptations of carbohydrate and creatine metabolism, as well as other HIF-1α -independent mechanisms, may enable cancers to survive hypoxic conditions despite anti-HIF1α therapy [47]. However, despite the lack of HIF-1α and reduced expression of glycolytic enzymes, cancer cells maintain glucose uptake through multiple mechanisms, including increased expression of glucose transporters and of creatine biosynthesis to support maintenance of ATP levels. Together with hypoxia, metabolic inhibition of 2-oxoglutarate-dependent dioxygenases controls HIF-1α stability and transcriptional activity. This pseudohypoxia is often adopted by cancer cells to sustain glycolytic metabolism for supporting biomass production for cell growth and proliferation. Under hypoxic/pseudohypoxic conditions, the levels of several metabolites, such as lactate, fumarate, and succinate, were increased, whereas citrate was decreased [45].

## 4. Cancer Cell Adaptation to Glucose Restriction

The existing evidence suggests that glucose deprivation restricts cancer cell proliferation more than that of healthy cells, and glucose concentration in the TME is lower if compared to that in normal tissues [48,49]. However, how cancer cells adapt to nutrient deprivation leading to metabolic flexibility in cancer progression remains to be fully understood. Lactic fermentation and OXPHOS [49] can both be operative in some cancer cells [50], with both contributing to cancer cell growth [19]. Several lines of evidence have highlighted the role of adaptive responses of cancer cells to nutrient deprivation. For example, the dependence of many tumor cell lines on OXPHOS in low-glucose conditions has been demonstrated in an RNA interference screen for metabolic liabilities with important differences in mitochondrial function between the most sensitive and the most resistant cancer cell lines to glucose limitation [51].

Pancreatic ductal adenocarcinoma (PDA) is a deadly cancer that exhibits drug resistance in a desmoplastic and nutrient-poor microenvironment. Several studies have investigated the effect of glucose or glutamine deprivation on PDA. Still, the effects on PDA growth and metabolism in this setting remain largely unknown. Tsai et al. reported the selection of cloned human PDA cells that survived and adapted to restricted levels of glucose and glutamine, showing that adapted clones increased growth in vitro and promoted tumor formation in vivo [11]. Adapted clones have shared transcriptional and metabolic programs, such as amino acid use for de novo glutamine and nucleotide biosynthesis. These results point to a non-genetic adaptation to nutrient deprivation in PDA and underscore glutamine synthetase 1 (GS1) as a possible therapeutic target in patients with pancreatic cancer [11]. Likewise, when two isogenic breast cancer cell lines (highly metastatic 4T1 and non-metastatic 67NR) were studied through dynamic magnetic resonance spectroscopy of ^13^C-isotopomers to identify differences in glucose and glutamine metabolism in response to metabolic and environmental stress, 4T1 cells had higher glycolytic and TCA cycle rates than those of 67NR cells and switched between glycolysis and OXPHOS when challenged with different extracellular conditions [14]. In isogenic cell lines derived from the same primary breast cancer, OXPHOS activity increased with metastatic potential, and TCA cycle restriction of succinate dehydrogenase (SDH) levels in 67NR cells resulted in succinate accumulation and OXPHOS inhibition. Indeed, environmental stress modulated the expression of SDH subunit A according to the metastatic potential of four isogenic cell lines. Furthermore, glucose-derived lactate production was more dependent on glutamine in cell lines with higher metastatic potential [14]. In addition, metastatic-forming 4T1 cells were more efficient at modulating metabolism in response to environmental stress compared to isogenic non-metastatic 67NR cells. Metabolic plasticity and adaptability are relevant to the metastatic breast cancer phenotype and could (1) provide a novel biomarker for early detection of this phenotype at the time of diagnosis and (2) suggest new treatment opportunities for metastatic breast cancer through mitochondrial metabolism [14]. Interestingly, long-term glucose deprivation does not affect equally different cell lines (i.e., the highly glycolytic breast cancer cells were the most affected) [48]. These data encourage the development of future trials in humans to find the appropriate timing of treatment (e.g., when cancer cells are most vulnerable) to introduce metabolic therapies. However, clinical studies of long-term glucose deprivation therapy are needed.

## 5. The Acidification of the TME

An inverse pH gradient, i.e., a lower pHe in an acidic microenvironment compared to the intracellular pH (pHi), has been suggested to exert a selective pressure that tumor cells must adapt to or die [52]. It has been hypothesized that acidic pHe regulates several functions in tumor cells, including activation of protective autophagy [53], acquisition of anoikis resistance [54], induction of the epithelial-to-mesenchymal transition (EMT) [55], promotion of local invasion [56], and enhancement of chemoresistance [57]. Recently, acidosis in the TME was recognized as a key factor in promoting cancer evolution and malignancy progression, rather than merely being an epiphenomenon [58]. Thus, acidosis of the TME has been proposed as an “obligatory step” for the high proliferative rate of cancer cells, which rely on glycolysis even in the presence of enough oxygen to sustain OXPHOS (i.e., near blood vessels), with a subsequent lactic acid release [59]. Mitochondrial respiration is also involved in acidification of the extracellular medium through spontaneous or enzymatic carbon dioxide hydration to generate carbonic acid [60]. Low pHe can be achieved due to lactic acid fermentation and increased excretion of protons and lactic acid under hypoxic conditions [61]. Cells can control their intracellular pH (pHi), but prolonged extracellular acidosis affects many aspects of cellular homeostasis, such as metabolism, signal transduction, and transcriptional activity [62,63]. A recently developed biophysical model suggests a role for carbonic anhydrase 9 (CA9) as a marker of tumor aggressiveness [64]. These data again strengthen the link between acidosis and hypoxia, suggesting that CA9 expression is regulated by hypoxia [65].

Recent results show that acidity leads to a reprogramming of a plastic tumor phenotype towards the expression of stem-related markers, high clonogenicity, and trans-differentiating ability [66], suggesting why extracellular acidosis can promote aggressive traits in cancer and contributing to tumor progression and metastasis. Furthermore, the acidosis-induced melanoma stem-like phenotype is reversible and associated with the induction of the epithelial–mesenchymal transition (EMT) [66]. Therefore, the use of drugs that control tumor extracellular acidosis may have important clinical implications for the treatment of aggressive solid tumors. A better understanding of the role of interstitial acidity and how tumor cells coordinate adaptive mechanisms in an acidified microenvironment is valuable for the improvement of therapeutic efficacy against solid cancers, particularly pancreatic ductal adenocarcinoma (PDAC) [67]. The acidification of the extracellular microenvironment is a slow and long-lasting process, which can explain the long latency period for tumor development, especially for initial primary lesions. However, studies to date have only examined solid tumor cells exposed to acidic pHe from minutes or hours to days or weeks [54,55]. Unlike in previous short-term studies, Wu et al. suggested that acidosis-mediated autophagy occurs primarily as an early stress response, but not in a later adaptation to prolonged extracellular acidification [67]. Thus, this study reveals a novel mechanism of early rapid response (extracellular-acidosis-induced autophagy) and late reversible adaptation (metabolic strategy) of PDAC cells to extracellular acidosis stress. This response is associated with an unusual pattern of mitochondrial network dynamics characterized by an early rapid response followed by a slow adaptation to persistent acidotic pHe stress [67]. They obtained a long-term acid-adapted cell population with significantly increased metastatic potentials and reported 34 acidic-pHe-related genes that were suggested as potential biomarkers/targets for the early diagnosis and treatment of PDAC [67], which, at least in part, may fill an existing knowledge gap in how solid tumor cells sense, respond, reprogram, and, finally, adapt to the persistent acid microenvironment. Several in vitro studies have reported that exposure of tumor cells to acidic pH increases the expression of pro-metastatic proteins, such as matrix metalloproteinases (MMPs), MMP-2, and MMP-9 [68]. The acidic TME creates a physiological barrier to cellular uptake of weak chemotherapeutic drugs that accumulate in the intracellular space. Moreover, the cellular uptake of some drugs is pH-dependent, so lowering the TME’s pH reduces chemotherapeutic efficacy with a physiological drug barrier described as “ion trapping”. This pH gradient can negatively affect the efficacy of weak base chemotherapy but is more suitable for weak acid therapy [69]. Interestingly, acidic pH and hypoxia promote migration of a mesenchymal triple-negative breast cancer cell line (SUM-159) [70].

As mentioned above, lactate was rediscovered as an important signaling molecule capable of determining the behavior and phenotype of tumors and tumor-associated cells [71]. For example, lactate impairs T-cell proliferation through reductive stress unrelated to microenvironmental acidification, thereby inhibiting NAD+, GAPDH, and PGDH responses and depriving proliferating T cells of glucose-derived serine. These results suggest a common mechanism between immune-privileged tissues and the TME, which share a high-lactate environment. NAD+ redox metabolism or lactate levels can be considered targets for stimulating immune modulation to enhance antitumor immunity or therapeutic immunosuppression [72]. In addition, therapeutic strategies for increasing low pHe, which is a common characteristic of solid tumors, are needed.

## 6. Cancer Cells Exploit “Atavistic Programs” for Their Adaptation and Plasticity to the Changed Microenvironment

### 6.1. “Atavistic Programs“ as a New Interpretive Framework in Cancer Biology

**Figure 1:**
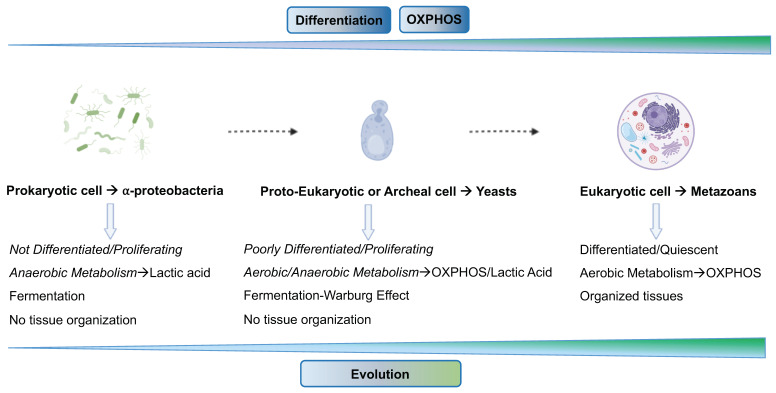
Schematic representation of evolutionary rewiring from prokaryotes to yeast to multicellular metazoans resulting in a phenotypic shift from undifferentiated, proliferative, and fermentative conditions to differentiated, quiescent, OXPHOS conditions. See the text for details. The figures were created with BioRender.

After Warburg [21,73], Szent-Gyorgy [74,75,76], and Weinhouse offered the first clues concerning the fermentative aspect of cancer cells about a century ago [77], recent evidence now supports the notion that most tumor cells depend on lactic acid fermentation rather than OXPHOS, independently of oxygen levels (i.e., the Warburg effect). The rewiring from OXPHOS to fermentation is also observed in forms of life such as prokaryotes (bacteria) and unicellular eukaryotes (yeasts) in particular environmental conditions [78]. The evolutive transition from bacteria to yeast to multicellular metazoans resulted in a phenotypic shift from an undifferentiated, proliferating, and fermenting condition to a differentiated, quiescent, OXPHOS condition (Figure 1).

The analogies between the yeast *Saccharomyces cerevisiae* and cancer cells have been underlined, and some enzymes, such as phosphofructokinase (PFK), pyruvate kinase (PK), lactate dehydrogenase (LDH), and succinate dehydrogenase (SDH), were proposed to drive the metabolic switch observed in yeasts [33]. Likewise, under hypoxic conditions, mammalian skeletal muscle goes through a temporary transition from OXPHOS to lactic acid fermentation, similarly to cancer cells, suggesting that “atavistic” metabolic processes are also conserved in multicellular eukaryotes [34].

**Figure 2:**
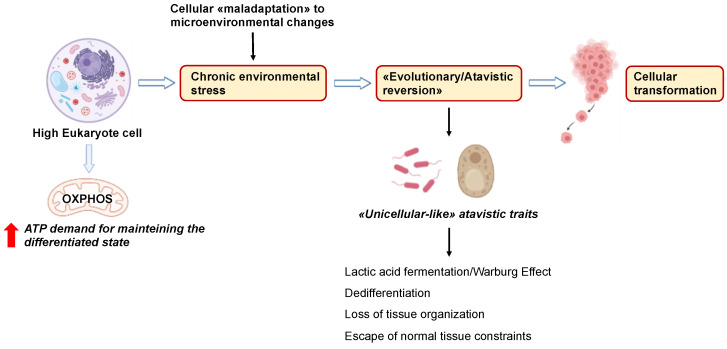
Schematic summary of the concept of carcinogenesis as an “atavistic regression” towards a pre-multicellular nucleated stage driven by chronic environmental stress. See the text for details. The figures were created with BioRender.

The correspondence among prokaryotes, unicellular metazoans (yeast), and cancer cells can be considered from the perspective of cancer biology (Figure 2).

In fact, the concept of cancer as an “atavistic regression” towards a pre-multicellular nucleated stage was proposed almost forty years ago by Setala, who addressed mitochondrial dysfunction as a stimulus for transformation and “de-evolution” caused by a “deficit in energy generation” [35]. Some authors have recently consolidated this idea, proposing cancer as an “atavistic” re-emergence and re-activation of genes of prokaryotic/unicellular eukaryote origin [36] that are not expressed in healthy multicellular eukaryotic cells [37]. Such “atavistic” modules would awaken due to the deregulation in the interactions between prokaryotic and eukaryotic genes, probably as a result of somatic mutations in regulatory genes at the boundary between unicellular and multicellular process genes [39]. This alteration could account for the adaptability and aggressiveness of tumor cells [38]. Notably, according to the “atavistic regression” theory, such “remerged” metabolic processes are more biologically robust than their normal eukaryotic ones [39]. This concept is important because current cancer therapies primarily target tumors’ strengths rather than their weaknesses.

This theoretical background led to the formulation of a new “Systemic–Evolutionary Theory of Cancer” (SETOC), which proposes that an alteration of the environmental milieu can “awaken” cellular processes characteristic of prokaryotes or unicellular eukaryotes [40,41,42]. Chronic inflammation, damage to the mitochondrial membrane, or modifications of its chemical composition by diverse chemical stressors may uncouple the balance between the two “coexisting systems” in favor of the “ancestral” programs, eventually boosting cancer development. This hypothesis encourages novel paradigms in cancer therapy that are more focused on systemic, process-founded strategies rather than on single molecular targets: a “process-based system pharmacology” approach [40].

### 6.2. Atavistic Regression as an Adaptive Response to the Changed Microenvironment

Recently, Yuichiro et al. suggested that “rather than thinking of the plasticity as an adaptation to a variable environment, it is better to consider plasticity as providing a foundation on which selection could act” [79]. This sentence refers to populations but can be considered as a key tenet of “genetic accommodation”, an adaptive genetic change. This can lead to genetic assimilation when a phenotypic trait, at first produced only in response to environmental changes, is formed even in the absence of such an influence, and thus, novel traits can be established or programmed to appear only under specific environmental conditions [80]. Thus, different abnormal phenotypes may result, which then become fixed due to the reoccurrence of extreme environmental conditions. Furthermore, polyphenic traits may evolve into robust traits if one developmental trajectory is favored over another [81]. In analogy, as proposed by the SETOC, changes in the cellular microenvironment (e.g., oxygen/nutrient deprivation, acidity, temperature) lead to adaptations that result in the uncoupling of the two endosymbiotic cell subsystems (the nucleocytoplasmic system and the mitochondria) toward ancestral or atavistic biologic functions and behaviors that are similar to those of unicellular organisms [16]. In other words, cancer cells become maladaptive after constantly trying to adapt to long-term damage. Maladaptation is sustained by “de-endosymbiosis”, which is the gradual breakdown of the two major cellular systems: nuclear–cytoplasmic (the primitive archaeal cell or proto-eukaryotic cell) and mitochondrial (primitive α-proteobacteria). Over selective pressure, the progressive de-endosymbiosis would lead to an evolutionary regression, with the appearance of phenotypic traits resembling those of unicellular organisms (“phylogenetic inversion”) and certain biological features of embryo development (“ontogenetic inversion”). This further implies a breakdown of multicellularity and the emergence of phenotypic features resembling those of unicellular organisms (“back to the roots”) and/or embryonic cells, conferring disorganization to cancerous tissues [16,17,39].

If we compare the “microenvironment” at the cellular level with the “macroenvironment” at the population level (phenotypic plasticity in both cases), we can say that adaptation leads to the developmental trajectories at the cell/tissue/organ and population levels, respectively, and we assume that cancer cells reactivate “old programs” (evolutionary throwback), which become “developmental trajectories” that make them more proliferative and more resistant; consequently, this phenotype becomes dominant. Indeed, non-genetic evolution is emerging as an important aspect in the fight against cancer drug resistance [4,39]. In addition, other evidence suggests that epigenetic modifications may happen independently of genetic changes, and they are thought to contribute to relapse [82]. We propose that the adaptation of cancer cells to changes in the microenvironment is a consequence of the plasticity of phenotypic responses, which can be maintained through evolutionary regression or adaptation to drug challenges. Importantly, both the adaptability to the external environment and “intracellular adaptability”, which tends to restore cellular homeostasis, cause the uncoupling of endosymbiosis [16,17]. This is an advantage for cells, allowing them to survive harsh conditions and become immortal. This gradual metabolic shift provides the basis for the development of new anticancer approaches [83].

In the light of the atavistic theory, the role of lactate in cancer transformation has been reconsidered [84,85]. Indeed, lactate causes acidification of the TME (as a consequence of the hypoxic state), suppresses antitumor immunity, fuels cell proliferation, and promotes angiogenesis during tumor growth, but also stimulates cell migration, metastasis, and release of tumor exosomes. In fact, we recently hypothesized that lactic acid fermentation could be considered a maladaptive mechanism and an evolutionary regression promoting drug resistance [39]. Furthermore, alterations in the TME can promote the formation of other oncogenic metabolites and induce protein modifications at the post-translational level, facilitating cellular adaptation to new environments [84,85]. Hence, targeting lactate production and transport may represent a promising approach for cancer therapy [39,86]. We also suggest that biological processes rather than single molecular targets (genes, proteins, metabolic enzymes, etc.) should be considered in cancer therapy in order to target the strength of cancer through a systems biology approach [39,83].

### 6.3. Microenvironmental Changes Deregulate Nucleo-Cytoplasmic Crosstalk during Tumorigenesis

It is well known that the metabolic state of a cell affects the exchange of information between the nucleus and cytoplasm. A specialized enzyme network catalyzed by adenylate kinase and/or creatine kinase is necessary for optimal crosstalk between the cytoplasm and nucleus [87]. Thus, energy communication is considered an important function of nuclear–mitochondrial crosstalk, and ATP is required for nuclear transport. Interestingly, inhibition of OXPHOS abolished nuclear transport, while inhibition of glycolysis decreased ATP production but did not affect transport [87]. In addition, some authors revealed a pathway for mitochondrial–nuclear signaling, demonstrating that some protein complexes, such as pyruvate dehydrogenase complex (PDC), which converts pyruvate into acetyl-CoA, translocate from the mitochondria to the nucleus via a still-unknown mechanism [88]. Whether this translocation is also followed by other mitochondrial matrix proteins and whether acetyl-CoA produced by nuclear PDCs can be used in other biochemical processes remains to be answered. Even so, studying the TME in which this translocation occurs may help to understand its mechanism. In addition, a non-canonical TCA cycle has recently been identified in the nucleus, linking metabolism to epigenetic regulation. Virtually all TCA-cycle-related enzymes (except succinate dehydrogenase) are present in the nucleus, catalyzing an incomplete TCA cycle that is similar to that found in cyanobacteria, where the main role is to produce/consume metabolic intermediates rather than energy generation [89]. This may support the atavism hypothesis. However, some recent results suggest that not only specific enzymatic steps but also TCA cycle subnetworks function in the mammalian nucleus [90]. In addition, some metabolic enzymes have noncanonical or nonmetabolic functions—the so-called ‘‘moonlighting’’ functions are involved in many cellular processes, including TME remodeling [91]. Our hypothesis can be further supported by the fact that prokaryotic cells are able to recognize nutrients and utilize them selectively, as well as to control their movements in the direction where nutrients are more promptly available. Furthermore, the behavior of prokaryotic cells is known for coordinating migration, chemosensing, and activation of targeted response systems to restore genome integrity in the presence of DNA damage [92]. Likewise, cancer cells can communicate information about lesions to nearby relatives, and the mechanisms behind this communication can provide important answers, including the formation of metastases.

### 6.4. Atavism and Adaptation in Cancer Drug Resistance and Metastasis

Drug-tolerant persister (DTP) cells are comparable to persister bacterial cells observed after antibiotic treatment [93]. Cancer DTPs occur at a low frequency in the tumor cell population and are characterized by reduced growth and altered metabolism, which increases drug tolerance. Such cells are genetically identical to the bulk tumor population, their resistance reverts upon removing the drug, and they develop in populations generated from single-cell clones, showing that persistence can be due to epigenetic regulation [4,94,95]. Importantly, non-genetic drug resistance is not restricted to a single cancer type or treatment, behaving as a general feature of cancer drug resistance. Despite the increasing number of publications on this topic, insufficient attention has been paid to methods of preventing non-genetic resistance and to developing specific therapeutic strategies targeting such resistance mechanisms [4,96]. In fact, therapeutic approaches should target not only molecular pathways, but also microenvironmental factors. A synergistic approach could ameliorate the adverse outcomes of many cancers. This also means that by changing microenvironmental conditions, we can change cancer cells’ response, taking the cancer cell population under control rather than using the “heavy artillery” to eradicate them [97]. Our research group recently demonstrated that the plant *Crithmum maritimum* effectively reduces the growth of hepatocellular carcinoma (HCC) cells by reverting the Warburg effect and bringing tumor cell metabolism closer to that of healthy hepatocytes [98,99,100]. Furthermore, we found that *Crithmum maritimum* can increase the drug sensitivity of HCC cells to sorafenib [101,102]. In addition, the antineoplastic effects of natural compounds [103,104,105], including their impact on the TME, have been reported, although more research is needed in this area [106,107].

Understanding cancer cell adaptations and metabolic reprogramming can also improve the knowledge in cancer cell biology by investigating our hypothesis that cancer cells revert to “atavistic” metabolic programs. However, our experimental work raises some questions that we have tried to answer. Are these processes of “retrieving” a consequence of both acute and chronic environmental change(s)? Well-designed, mechanistic experiments can help us explain not only why it occurs, but also when (after acute or chronic exposure to harsh conditions) and how non-genetic responses (such as drug resistance) occur. We believe that understanding the basis of these processes is important for targeting the metabolic requirements [4] that characterize the different steps of tumor formation (i.e., initiation, progression, and metastasis) [108]. This is more related to the metastatic processes, as the composition of the TME directly affects the success of metastasis [109], and metabolic reprogramming is a hallmark of cancer metastasis [110], while the noninvasive analysis of the cancer metabolic phenotype can prove useful in obtaining information about tumor metastatic propensity [14]. Whereas several investigations concentrated on finding gene signatures associated with the pathophysiology of metastases (the theory of “seed and soil”) [111], there is an increasing number of theories indicating that the metastatic process is independent of cell relocation and happens at a distance from primary cancer cells through the uptake of circulating cancer factors by primed cells in the target organs (horizontal transfer of malignant traits) and, subsequently, resulting in transformed phenotypes similar to that of the donor primary cancer cell [112,113]. In both cases, microenvironment-induced changes should not be underestimated [114], since the TME is a milieu where the cancer-associated network drives metabolic reprogramming, leading to cancer onset and progression, as well as horizontal transfer of neoplastic traits, but also to the development of therapeutic resistance due to the release of extracellular vehicles (EVs) [115]. Indeed, recently, it has been described that EVs delivered by human cancer cells (known to contain genetic material and signaling molecules) may be up-taken by onco-suppressor-mutated cells (OMCs), which become malignant with the phenotype compatible with those of the cancer cells from which the EVs were derived [116]. In addition, EV release in cancer has also been described as an adaptation to a microenvironmental selection pressure that further promotes the survival of cancer [117]; thus, the release of EVs may occur in (or be controlled by) specific microenvironmental conditions before circulating oncogenic factors are present in circulation. On the other hand, the cargo of tumor-derived EVs can remodel the tumor microenvironment [118]. Indeed, long-term exposure to a hostile microenvironment may promote disease recurrence after surgical intervention, but it may also be predictive when the disease has a silent course [119,120].

Hence, targeting microenvironmental conditions may have an important impact on prevention, treatment, and, especially, the course of the disease and its recurrence. Furthermore, its combination with novel biological platforms for cancer screening that can detect dysplastic lesions, premalignant lesions, and cancer in situ may have a major impact on cancer prevention and management [121]. On the other hand, in addition to the identification of the specific circulating factors, elucidating the mechanisms underlying the progressive uncoupling of the endosymbiotic subsystems in the cells might also help in finding specific genes/enzymatic pathways that could be targeted (e.g., cytochrome c oxidase 1 and 2, COX-1, and COX-2), which may further contribute to the higher precision of widely used conventional liquid biopsies, but might also help in elucidating cancer specificity [122]. This metabolic plasticity, also known as a “hybrid” metabolic state, permits the production of energy through multiple metabolic pathways that support tumor metastasis and treatment resistance. Whether specific microenvironmental conditions drive this movement is another challenging question. The importance of the microenvironment is further supported by the fact that the embryonic microenvironment is known to be non-permissive for tumor development, and one study recently showed that human-embryonic-stem-cell-derived exosomes have a crucial role in tumor suppression [123]. Consequently, complex cancer networks and signaling can be controlled by targeting metabolic processes [83]. This also means that from a metabolic point of view, an individualized approach is important, so concomitant diseases and treatments should be carefully considered when making treatment decisions.

## 7. Targeting Energy Metabolism and the Microenvironment in Cancer with Different Strategies

Regarding therapeutic approaches that target cancer energy and metabolism, inhibitors of the glycolytic pathway have been developed, and this approach led to the formulation of some drugs that increase mitochondrial respiration, which was suggested to be involved in inducing tumor cell arrest and death [124]. It is worth noting that previous studies in this area mainly focused on glycolysis, whereas an approach targeting OXPHOS has recently emerged [125]. Some cancer cells, such as those in leukemia, lymphoma, and pancreatic ductal adenocarcinoma, rely on this pathway [126], and under specific microenvironmental conditions, this metabolic shift allows cancer cells to survive anticancer treatments. In addition, it has been shown that cancer stem cells can be in a quiescent (dormant) or self-renewing state, exhibiting enhanced OXPHOS [127,128]. While pharmacological ETC inhibitors reduce mitochondrial respiration and induce apoptosis in cancer cells, cancer cells modulate their metabolism, shifting to a glycolytic mode and resulting in a drug-resistant phenotype [129]. We recently showed how to overcome sorafenib resistance in hepatocellular carcinoma by switching glycolysis to OXPHOS [130]. This could support the use of combination therapies to enhance the efficacy of conventional therapies, as well as the development of new rationally designed combinations [131]. ETC inhibitors can be used in specific situations, such as targeting TME subpopulations with limited access to glucose, cancer cells that are highly dependent on OXPHOS [132], or quiescent cancer cells that rely on OXPHOS metabolism for tumor recurrence [133]. Therefore, addressing tumor metabolism in combination with currently available therapies may confer therapeutic advantages.

Changes in the TME lead to metabolic reprogramming of tumors associated with molecular adaptations. Several conditions should be considered, such as crosstalk between cancer and stromal cells, cancer cell adaptability/plasticity, and the predominance of less prevalent phenotypes after antineoplastic therapy. Consequently, the central role of mitochondria in tumor development and flexibility is becoming clearer and increasingly supported by different research groups. Metabolic flexibility mediated by mitochondrial function supports cancer cell survival and proliferation [134]. Thus, new therapeutic approaches targeting metabolic targets, including mitochondria-related targets, are under investigation [20]. Mitochondrial metabolism has been suggested as a new strategy for metastatic breast cancer [14], and the authors propose that metabolic plasticity and adaptability are more important in this type of cancer phenotype than rapid cell proliferation alone. Notably, impaired glucose utilization is a potential cancer biomarker and a challenge for future studies [51].

Controlling the microenvironmental conditions and the resulting tumor adaptations can provide tools for improving the success of anticancer therapies. This is especially important for preventing cancer recurrence after surgical intervention and during drug therapy and/or cellular adaptation and resistance to treatment [96]. More recently, the critical role of amino acid (AA) metabolic reprogramming in cancer cells and the role of a supportive TME in driving resistance to anticancer therapies was highlighted [135]. Importantly, the modulation of plasma AA levels was achieved through pharmacological or dietary interventions, supporting the use of natural products as supplements. However, the decision to modify the diet is critical to successful treatment and must be chosen according to the specific characteristics of the tumor. Interestingly, the authors showed that cancer cells’ specific sensitivity to dietary modulation may be provided by the individual genetic alterations and the metabolic profile of the parental tissue. The use of compounds with antineoplastic effects targeting metabolites such as pyruvate, citrate, and glutamine synthetase inhibitors has been considered as a therapeutic strategy. Furthermore, some of these metabolites, as well as mutations in the related enzymes, have been recognized as markers of malignancy (e.g., high lactate levels) [45]. Hence, studying these mutations may open the way for personalized diagnostics. Moreover, Loong et al. emphasized the therapeutic potential of targeting fucosyltransferase 1 (FUT1) and/or their glycoprotein targets to treat nutrient-deficient HCC and other tumors [136], which may also represent good prognostic markers.

Existing evidence suggests that targeting HIF-1α is not sufficient to disrupt the metabolism of cancer cells [47], which highlights the need for further investigation of anti-HIF-1 drugs, including their combination with other therapeutic approaches [137]. Advanced in vitro models represent an alternative approach to modeling the TME by using technologies such as microfabrication. Advanced model systems, such as microfluidic tumor-on-a-chip models, provide a promising platform for modeling the physicochemical conditions of the TME in a controlled system, as recently described [5]. These systems, which are designed to maintain tumor cells in an environment similar to in vivo conditions, can reproduce the gradients of oxygen, nutrients, and endogenous chemokines and can mimic cell proliferation. Furthermore, establishing mechanistic cues in such models, such as extracellular matrix properties that influence cell behavior, facilitates the analysis of the dynamic interactions between different conditions and their potential synergistic effects on tumor cells [5]. Recently, a high-throughput microfluidic platform with a controlled oxygen environment was employed to monitor mesenchymal migration under hypoxic conditions. Authors suggest that the efficiency of anti-metastatic drugs is increased under hypoxic conditions combined with a slightly alkaline TME [70].

As for the acidic TME, low-pH-activated micellar systems have been shown to be able to enter inside solid tumors [138,139]. Several results have shown that acidic pH in combination with an oxygen gradient can promote tumor cell migration [70]. The ability of this device to manipulate the TME in terms of oxygen and pH provides the opportunity to analyze the effects of different conditions at the same time. However, other novel approaches to overcoming low pH and drug resistance are needed to improve the therapeutic efficacy of current and future compounds. A decrease in extracellular pH suppresses glycolysis in T cells, induces reductive stress during the hypoxia, and stimulates inhibitory immune checkpoint molecules [140]. Overcoming these metabolic effects will require innovative strategies for selectively targeting tumor cells, thereby sustaining T-cell function, or the use of synergistic approaches to address both factors [140].

Finally, a “negotiation strategy” (based on negotiation rather than eradication) has been proposed to set up cancer treatment [141], including the use of buffers that will directly neutralize acid, that is, the development of acid-activatable drugs (i.e., nanomedicines) and the inhibition of metabolic processes causing acidosis [58]. Although its importance has not been established, acid adaptation has also been associated with the release of EVs from tumor cells under hypoxic conditions, thereby increasing and stabilizing exosomal RNA and protein content [142,143]. Compared to transient hypoxic conditions [144], acidification of the TME may remain stable due to aerobic glycolysis, which is associated with drug resistance, and new drugs are needed to address this condition [145]. Notably, some specific systems have been designed to respond to the TME, especially in fluorescence-imaging-guided cancer therapy [146,147], but such drug delivery systems with TME-responsive properties remain challenging. Further studies are needed to understand the variation of exosome content in different TMEs and how they could be a tool for the delivery of therapeutic molecules [137,148,149]. However, a recent pilot study demonstrated that artificial intelligence algorithms coupled with time-resolved fluorescence correlation spectroscopy power spectra can precisely distinguish complex patient-derived EVs from different cancer samples of distinct tissue subtypes [150].

The flexible utilization of nutrients by cancer cells is not only beneficial for tumor evolution, but the efficacy of antimetabolite drugs may also be affected [49]. In addition, blocking one metabolic pathway leads to an adaptive response in cancer cells, while simultaneously targeting multiple metabolic pathways may result in an efficacious synergistic response [151]. Therefore, future therapeutic approaches should also include inhibition of signaling pathways that support adaptation or are involved in metabolic flexibility, as well as targeting mitochondrial metabolic and apoptotic effects. A combination of metabolic therapies that inhibit the glycolysis and OXPHOS pathways may be more efficacious in the treatment of aggressive metastatic breast cancer [14]. Recently, the spare respiratory capacity (SRC) has been suggested as a parameter for determining mitochondrial reserve (the difference between basal respiration and respiration at its highest level), as it characterizes the mitochondrial capacity to meet extra energy requirements in response to acute cellular stress or heavy workload, thereby avoiding an ATP crisis. Thus, this reflects “healthy” mitochondria. Furthermore, this determines the metabolic bioenergetic signature and predicts the resistance of untransformed cells to stress and the aggressiveness of cancer cells, including drug resistance [152].

Increasing our knowledge of the molecular pathogenesis of cancer may be one way to personalize cancer care. However, despite the availability of many therapies targeting specific molecules essential for tumor development, treatment resistance and disease recurrence frequently occur, especially in later stages of the disease. Understanding the mechanisms that regulate plasticity will provide insight into how disease evolves [79], which may be particularly important because cellular plasticity enables cancer to evade immunity. Thus, these processes must be understood collectively, rather than in isolation. This will facilitate the development of innovative epigenetic therapies and therapeutic tools for improving outcomes for cancer patients. In addition, metabolic differences and the possibility of assessing adaptability in living cells open the field to identify new biomarkers, particularly for the early detection of metastatic phenotypes. Combining nutraceuticals with drugs may be beneficial in reducing drug resistance [102], but also in maintaining microenvironmental conditions that may lead to relapse. However, many questions remain unanswered. Are the mechanisms and/or phenotypes of drug-resistant populations different in different tissue microenvironments in the same host? Can we identify different phenotypic subpopulations with different therapeutic vulnerabilities? This will help us in predicting the future behavior of individual cancers and, in particular, which drug resistance evolutionary path they would take. Likely, most of them could be addressed by the application of technologies applied to dissect intratumor heterogeneity (genetic and non-genetic) in 4D (spatial and temporal) space and at the cell level, as well as innovative models that better approximate the complexity of cancer [3]. In addition, there is a need to develop tools that enable the simultaneous study of the (epi)genome and transcriptome.

## 8. Discussion and Future Directions

Metabolism-based single-target approaches have shown some criticality for what concerns efficacy and side effects. Therefore, a synergistic approach targeting multiple metabolic pathways or processes can prove useful and efficacious in improving efficacy and lowering dosages and toxicity. As an example, plant extracts, which include a blend of metabolites, can be considered an effective opportunity to target different metabolic pathways at the same time. In this regard, plant extracts have not received much consideration from this perspective, but there are still some studies reporting significant effects on different tumors, which may be due to the synergistic targeting of multiple pathways [153,154,155,156,157]. Notably, we recently investigated *Crithmum maritimum*, an edible wild plant that grows naturally along the Mediterranean and Atlantic coasts. We screened the chemical composition and biological effects of *Crithmum maritimum* harvested along the Apulian coast, and we found that ethyl acetate extract of *Crithmum maritimum* inhibits HCC cell growth by a synergistic inhibition of several metabolic pathways involved in HCC growth, such as lactic acid fermentation/the Warburg effect, lipid and cholesterol metabolism, and amino acid metabolism. *Crithmum maritimum* ethylacetate extract is rich in falcarindiol and chlorogenic acids, which have anticancer properties [99]. In addition, we found that *Crithmum maritimum* promotes OXPHOS and inhibits LDHA activity and, hence, lactate production [98], and it increases the expression of markers typical of healthy hepatocytes [101]. Therefore, *Crithmum maritimum* can be considered a model of how to improve traditional medical treatment of cancer [102].

After decades of genetic and molecular-biology-based cancer research, there is now a renewed interest in the role of metabolism in promoting and supporting carcinogenesis. Several biological pathways have been investigated, and many enzymes have been suggested as potential therapeutic targets. Even so, the complex crosstalk between the different pathways imposes a more complex, multitarget, process-directed approach, which is directed at targeting more processes at the same time to reduce tumors’ “escape” strategies. Targeting single enzymes involved in the regulation of metabolic pathways was proposed and tried, but so far, the results obtained are not convincing in terms of efficacy and tolerability, which is probably due to the intrinsic pleiotropic capacity of tumor cells to bypass a single inhibitory stage. Therefore, a cooperative multi-objective/multi-process approach can be employed. In this regard, plant extracts may be considered as a valuable option due to their blended composition, which can target several processes at the same time. Coupled with this approach, selective inhibition of specific metabolism-controlling receptors may provide a supplemental option, as described above for LPAR6 [130,158].

## 9. Conclusions

**Figure 3:**
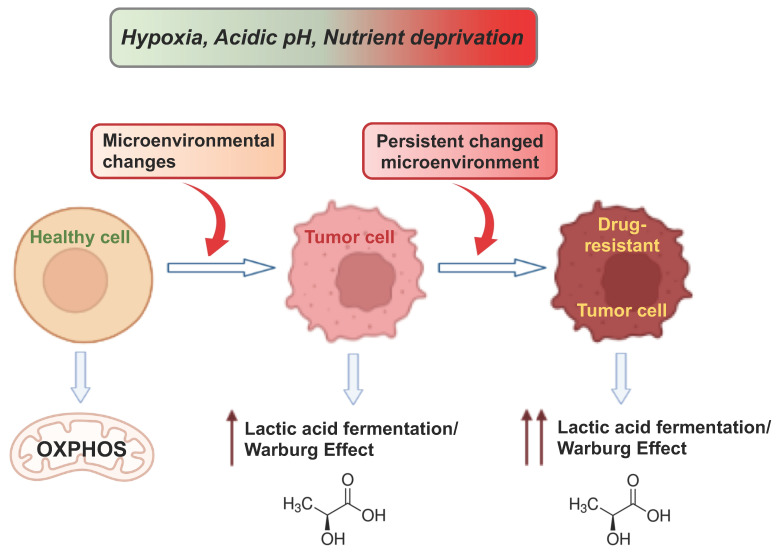
Schematic representation of the links between microenvironmental stress, metabolism, tumorigenesis, and drug resistance. See the text for details. The figures were created with BioRender.

Dysregulation of cellular metabolism is an important driver of tumorigenesis and drug resistance [159,160,161,162]. The balance between glycolysis and OXPHOS is a central aspect, and the mechanisms by which this is regulated are still not fully understood. We propose that the multifaceted interplay between tumor cell metabolism and drug resistance is relevant and that the balance between OXPHOS and fermentative glycolysis is central for cellular behavior during neoplastic transformation, as well as for the acquisition of drug resistance by tumor cells. In addition, we believe that regulating the balance between OXPHOS and lactic acid fermentation, promoting OXPHOS, and inhibiting fermentation can effectively reduce tumor invasiveness and drug resistance. In this regard, we recently demonstrated that inhibition of OXPHOS in favor of lactic acid fermentation triggers drug resistance in HCC [130]. A deeper understanding of how metabolic and microenvironmental processes drive cellular adaptation is crucial. In this regard, it is worthy to emphasize that, recently, an in vitro reproduction of a mix of harsh microenvironmental conditions was able to induce a Warburg phenotype in breast cancer [163]. However, the biological mechanisms by which normal cells transform and acquire neoplastic phenotypes have not been fully elucidated. Elucidating these mechanisms is an important step toward understanding how cells behave and how they begin to malfunction. Figure 3 summarizes the links between microenvironmental stress, metabolism, tumorigenesis, and drug resistance.

In conclusion, we believe that approaches to modulating the metabolism of fermenting tumor cells may offer opportunities to reduce cancer drug resistance. In particular, we propose activation of OXPHOS and suppression of the lactic acid fermentation/the Warburg phenotype to reduce tumor growth and drug resistance in combination with pro-differentiation strategies. This “metabolic approach” should be independent of the genetic context of different tumor, making it an advantage for therapeutic purposes. An integrative perspective based on selective interventions in the TME and tumor cell metabolism can provide new efficacious opportunities for cancer diagnosis and therapy and can reduce drug resistance, recurrence, and side effects.

## Data Availability

The data can be shared up on request.
